# Codon usage characterization and phylogenetic analysis of the mitochondrial genome in *Hemerocallis citrina*

**DOI:** 10.1186/s12863-024-01191-4

**Published:** 2024-01-13

**Authors:** Kun Zhang, Yiheng Wang, Yue Zhang, Xiaofei Shan

**Affiliations:** 1 College of Agriculture and Life Sciences, Shanxi Datong University, Datong, Shanxi China; 2 State Key Laboratory of Vegetable Biobreeding, Tianjin Academy of Agricultural Sciences, Tianjin, China; 3 Key Laboratory of Organic Dry Farming for Special Crops in Datong City, Datong, Shanxi China

**Keywords:** *Hemerocallis citrina* Baroni, Mitochondrial genome, Codon usage bias, Phylogeny

## Abstract

**Background:**

*Hemerocallis citrina* Baroni is a traditional vegetable crop widely cultivated in eastern Asia for its high edible, medicinal, and ornamental value. The phenomenon of codon usage bias (CUB) is prevalent in various genomes and provides excellent clues for gaining insight into organism evolution and phylogeny. Comprehensive analysis of the CUB of mitochondrial (mt) genes can provide rich genetic information for improving the expression efficiency of exogenous genes and optimizing molecular-assisted breeding programmes in *H. citrina*.

**Results:**

Here, the CUB patterns in the mt genome of *H. citrina* were systematically analyzed, and the possible factors shaping CUB were further evaluated. Composition analysis of codons revealed that the overall GC (GCall) and GC at the third codon position (GC3) contents of mt genes were lower than 50%, presenting a preference for A/T-rich nucleotides and A/T-ending codons in *H. citrina*. The high values of the effective number of codons (ENC) are indicative of fairly weak CUB. Significant correlations of ENC with the GC3 and codon counts were observed, suggesting that not only compositional constraints but also gene length contributed greatly to CUB. Combined ENC-plot, neutrality plot, and Parity rule 2 (PR2)-plot analyses augmented the inference that the CUB patterns of the *H. citrina* mitogenome can be attributed to multiple factors. Natural selection, mutation pressure, and other factors might play a major role in shaping the CUB of mt genes, although natural selection is the decisive factor. Moreover, we identified a total of 29 high-frequency codons and 22 optimal codons, which exhibited a consistent preference for ending in A/T. Subsequent relative synonymous codon usage (RSCU)-based cluster and mt protein coding gene (PCG)-based phylogenetic analyses suggested that *H. citrina* is close to *Asparagus officinalis*, *Chlorophytum comosum*, *Allium cepa*, and *Allium fistulosum* in evolutionary terms, reflecting a certain correlation between CUB and evolutionary relationships.

**Conclusions:**

There is weak CUB in the *H. citrina* mitogenome that is subject to the combined effects of multiple factors, especially natural selection. *H. citrina* was found to be closely related to *Asparagus officinalis*, *Chlorophytum comosum*, *Allium cepa*, and *Allium fistulosum* in terms of their evolutionary relationships as well as the CUB patterns of their mitogenomes. Our findings provide a fundamental reference for further studies on genetic modification and phylogenetic evolution in *H. citrina*.

## Background

The codon represents the fundamental connection between genes and proteins when deciphering genetic information. In the 64 standard genetic codes, there are 61 sense codons encoding 20 types of amino acids, and the remaining three are translation termination signals. Compared to the number of codable amino acids, the excess of possible nucleotide triplets results in a redundancy of the genetic code. Indeed, apart from tryptophan and methionine, which are encoded by a single codon, all other gene products are translated by two to six different triplets, a phenomenon defined as codon degeneracy [1]. Multiple codons that are decrypted into an identical amino acid are referred to synonymous codons, which are not uniformly utilized during protein synthesis in many organisms [2]. This species preference for certain codons, termed codon usage bias (CUB), is a consequence of the optimization of the deciphering strategy and plays an imperative role in the gene expression regulation [3, 4]. Information on CUB can provide important insights into exogenous gene expression [5], gene function prediction [6], genetic divergence assessment [7], and organism evolution exploration [8] and can contribute to revealing the molecular mechanisms underlying the environmental adaptation of various species [9].

The degree of CUB divergence differs widely across species, genes, and even within an individual gene [10, 11]. Causes for the existence of CUB in organisms are diverse and complicated. In the process of long-term evolution, CUB deviations are primarily driven by natural selection, directional mutation, and random genetic drift [12]. With the continuous progress of genome sequencing and bioinformatics, additional factors of complexity involved in CUB have been established over the last few decades, including genome size [13], gene expression pattern [14] and degree [15], gene length [16], efficient gene translation initiation [17], tRNA abundance [18] and interactions [19], synonymous substitution frequency [20], and mRNA folding [21], among others. Moreover, the patterns of CUB appear to be related to phylogenetic relationships, i.e., the more closely phylogenetically related species tend to share a more similar CUB pattern [22]. Given all of this, CUB is highly complex, and understanding it is challenging when considering the difficulty in determining the relative effect of the various factors. Much more detailed analyses of this fascinating phenomenon are needed to broaden our understanding of its biological implications and applications.

Mitochondria (mt) are semiautonomous energy-producing eukaryotic organelles that drive oxidative phosphorylation for energy metabolism [23]. Ordinarily, plant mt genomes (mitogenomes) exhibit more complex features compared with both their counterparts in animals and the conserved plastid genomes of plants [24]. Ongoing advances in sequencing and assembly technologies have significantly promoted the complete sequencing of mitogenomes in land plants, but nevertheless, there is a requirement for more available data to gain more refined knowledge of plant mitogenomes. The analysis of codon preference in plant mitogenomes is of great significance for studying the genetic patterns, phylogenetic relationships, and evolution of their mtDNA. Although the research of CUB in plant mitogenomes has made continuous progress [25–27], it has not been addressed more extensively and intensively like its equivalent nuclear and plastid genomes.

*Hemerocallis citrina* Baroni belongs to the Asphodelaceae family and is a popular perennial herbaceous plant widely cultivated across Asia for food nutrition [28], medicinal properties [29], and landscape beautification [30]. The immature flower buds are generally processed into dried vegetables with high nutraceutical value. *H. citrina*, also respected as the mother’s flower, has a long cultivation history and unparalleled cultural significance in China [31]. Recent studies have demonstrated that *H. citrina* is rich in flavonoids, polyphenols, alkaloids, and anthraquinones [29, 30], making it a potent medicine for anti-inflammatory, antidepressant, and antioxidant uses. The successive acquisition of sequence information for the chloroplast (cp) [32] and nuclear [33] genomes symbolizes the considerable progress of *H. citrina* genomics research in recent years. Our team adopted a strategy of integrating Oxford Nanopore long-read and Illumina short-read sequencing to complete the sequencing, assembly, and annotation of the *H. citrina* mitogenome [34]. However, systematical analysis on the CUB of the mitogenome has not been performed in *H. citrina.* The knowledge gained from CUB research provides useful clues for improving the expression level of exogenous genes and optimizing molecular-assisted breeding programmes in *H. citrina*. Consequently, it is particularly significant to analyze the CUB patterns and further evaluate the evolution and phylogeny of *H. citrina*, considering its tremendous economic benefits and various utilities. In this research, we conducted comprehensive analysis of the CUB of mt genes in *H. citrina*. We investigated the codon composition characteristics and usage patterns and evaluated the factors that influence CUB. Furthermore, relative synonymous codon usage (RSCU)-based cluster and mt protein coding gene (PCG)-based phylogenetic analyses were performed to advance the understanding of the evolution and phylogeny of *H. citrina*. The results derived from this work may help to facilitate the mt gene utilization, genetic improvement, and molecular breeding of *H. citrina*.

## Results

### Codon composition of the *H. citrina* mitogenome

The final 28 protein coding sequences (CDS) of the mitogenome in *H. citrina* were available for codon usage analysis. The overall GC content of the whole mitogenome (GCall) was estimated at 43.59%, and the frequency of GC at each codon position (GC1, GC2, and GC3) was lower than 50% without exception (Table 1). Although the percentage of the GC composition in each gene was slightly different, the content order ranking of GC1 > GC2 > GC3 was highly consistent (Table 2). Furthermore, the average GC composition at the third position of synonymous codons (GC3s) of the CDS was lower than 50%, and the percentage of each individual base at the synonymous site (A3s, C3s, G3s, and T3s) conformed to the order ranking of T3s > A3s > G3s > C3s (Table 1), indicating that the codons of the *H. citrina* mitogenome tend to end in A/T.

**Table 1:** Codon composition parameters of the mitogenome in *H. citrina*

Codon counts	Base composition at the third position of the synonymous codon/%				GC content/%					ENC
	T3s	C3s	A3s	G3s	GC1	GC2	GC3	GCall	GC3s	
8850	40.32	22.59	36.02	23.05	48.67	43.05	39.05	43.59	36.04	53.89

**Table 2:** Codon characteristic parameters of mt coding genes in *H. citrina*

Gene	Codon counts	GC1(%)	GC2(%)	GC3(%)	GCall(%)	CAI	CBI	Fop	ENC
*atp1*	510	57.45	42.55	34.90	44.97	0.17	-0.10	0.35	52.75
*atp4*	195	44.62	43.08	37.95	41.88	0.16	-0.08	0.36	59.92
*atp6*	248	44.76	37.90	31.85	38.17	0.15	-0.18	0.29	50.34
*atp8*	281	45.20	33.45	43.42	40.69	0.17	0.01	0.41	58.37
*ccmB*	207	45.41	43.48	34.30	41.06	0.17	-0.07	0.35	44.91
*ccmC*	273	47.25	48.35	35.90	43.83	0.17	-0.01	0.39	49.88
*ccmFc*	449	48.78	44.77	42.32	45.29	0.14	-0.10	0.35	57.34
*ccmFn*	614	50.16	48.86	42.02	47.01	0.16	-0.05	0.38	57.28
*cob*	390	50.26	41.54	34.62	42.14	0.16	-0.12	0.32	56.44
*cox1*	528	48.30	45.45	36.74	43.50	0.19	-0.03	0.39	53.89
*cox2*	273	52.01	39.56	33.33	41.64	0.20	-0.07	0.36	49.39
*cox3*	266	52.26	45.11	35.34	44.24	0.20	-0.04	0.38	56.00
*matR*	673	53.94	43.39	56.76	51.36	0.15	0.02	0.42	57.49
*nad2*	182	40.66	38.46	36.81	38.64	0.16	-0.16	0.30	54.26
*nad3*	119	44.54	46.22	36.97	42.58	0.19	-0.11	0.33	48.69
*nad4*	496	46.57	43.95	36.69	42.41	0.16	-0.06	0.36	52.72
*nad5*	482	44.40	46.06	38.38	42.95	0.17	-0.11	0.35	56.49
*nad6*	232	46.55	41.81	44.40	44.25	0.14	-0.04	0.36	58.91
*nad7*	395	56.46	46.08	30.89	44.47	0.17	-0.05	0.36	49.56
*nad9*	191	51.83	42.41	32.46	42.23	0.21	-0.06	0.39	54.87
*rpl5*	196	49.49	36.22	41.84	42.52	0.17	-0.09	0.37	56.43
*rps1*	167	47.90	38.92	41.92	42.91	0.18	-0.10	0.35	49.21
*rps2*	232	42.67	40.95	36.21	39.94	0.17	-0.12	0.35	55.45
*rps3*	562	44.31	40.21	43.77	42.76	0.15	-0.07	0.38	60.01
*rps4*	345	41.74	40.58	38.55	40.29	0.12	-0.03	0.39	55.18
*rps12*	126	56.35	47.62	30.16	44.71	0.15	-0.02	0.40	55.71
*rps13*	117	50.43	40.17	29.91	40.17	0.16	-0.12	0.35	39.34
*rps14*	101	42.57	46.53	36.63	41.91	0.16	-0.08	0.35	51.37

In the analysis of 28 CDS in the mitogenome, a total of 8850 codons were also obtained (Table 1), involving all 64 types of codons. The codon number of the mt genes in *H. citrina* varies greatly, ranging from 101 in *rps14* to 673 in *matR* (Table 2). The effective number of codon (ENC) values range from 39.34 to 60.01, with an average of 53.89, exceeding 50 in the mitogenome. All of the genes had ENC values greater than 35, and up to 75% of them had high (> 50) ENC values, indicating fairly weak CUB in *H. citrina*. In addition, the codon adaptation index (CAI) values of the mt genes ranged from 0.12 to 0.21, with a mean value of 0.17, far less than 1. The values of codon bias index (CBI) and frequency of optimal codons (Fop) were clustered around − 0.18–0.02 and 0.29–0.42, respectively. In conclusion, the above results suggest that both codon bias and mt gene expression are relatively low in *H. citrina*.

### Correlation analysis between CUB parameters

**Fig. 1:**
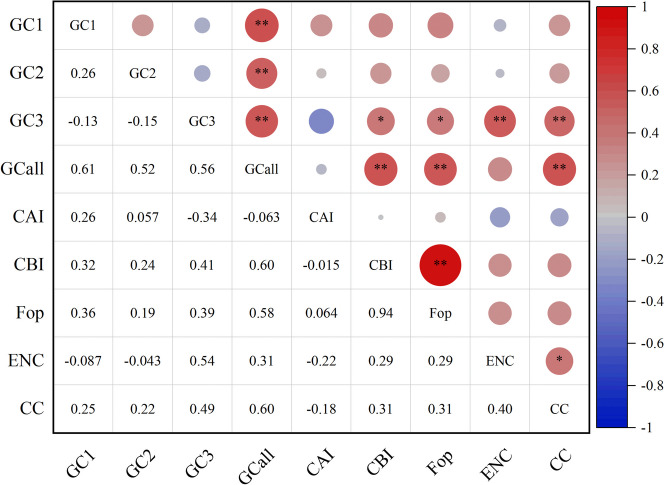
Correlation analysis of codon parameters in the *H. citrina* mitogenome. *, ** indicate correlations significant at the 0.05 and 0.01 levels, respectively

To reveal the role of the composition properties in CUB, Pearson’s correlation analysis was conducted between the important indices of codon usage. The results displayed a significantly positive correlation between GCall and GC1, GC2, and GC3 (*P* < 0.01, Fig. 1), indicating an overall strong correlation of the composition among the three codon bases in the mitogenome. The ENC value had a significantly positive correlation with GC3 (*P* < 0.01), implying that the base composition of the synonymous site has a crucial impact on CUB. Simultaneously, ENC positively correlated with the codon counts (CC) (*P* < 0.05), which elucidates that gene length also contributes greatly to codon bias. Further, it was found that CBI and Fop were significantly correlated with GCall (*P* < 0.01) and with GC3 (*P* < 0.05), indicating that GCall is another major factor that affects CUB.

### Cause analysis of codon usage preference

**Fig. 2:**
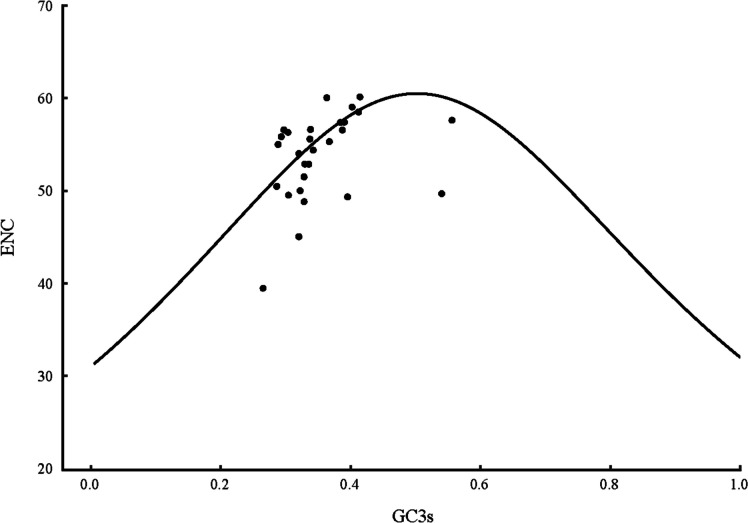
ENC-plot analysis of the *H. citrina* mitogenome

**Fig. 3:**
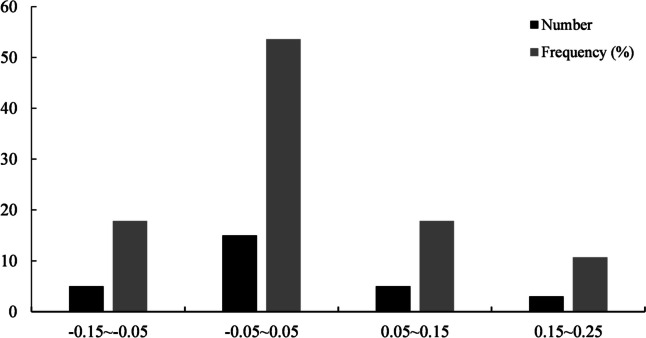
Distribution of ENC frequency of the *H. citrina* mitogenome

For purpose of understanding whether the G + C mutation bias influences the CUB of *H. citrina*, the ENC for genes were mapped against the GC3s. The ENC-plot of *H. citrina* is displayed in Fig. 2. Only a few genes approached the solid curve, inferring that compositional mutation plays a significant role in CUB. However, most of the genes were scattered on both sides away from the standard curve, implying that natural selection has also shaped the CUB patterns. Besides, to better estimate the difference in ENC values, the ENC frequency distribution of the current genes was analyzed. The ENC ratio varied from − 0.15 to 0.25 (Fig. 3). Among the 28 mt genes, 19 (67.86%) had an ENC ratio greater than 0, reflected by these genes being distributed below the standard curve. Additionally, 15 genes (53.57%) were distributed within the range of -0.05–0.05 and had slight differences between the actual and expected ENC values. These results further demonstrate that the CUB patterns of the *H. citrina* mitogenome might be shaped by the joint effects of natural selection and mutation pressure.

**Fig. 4:**
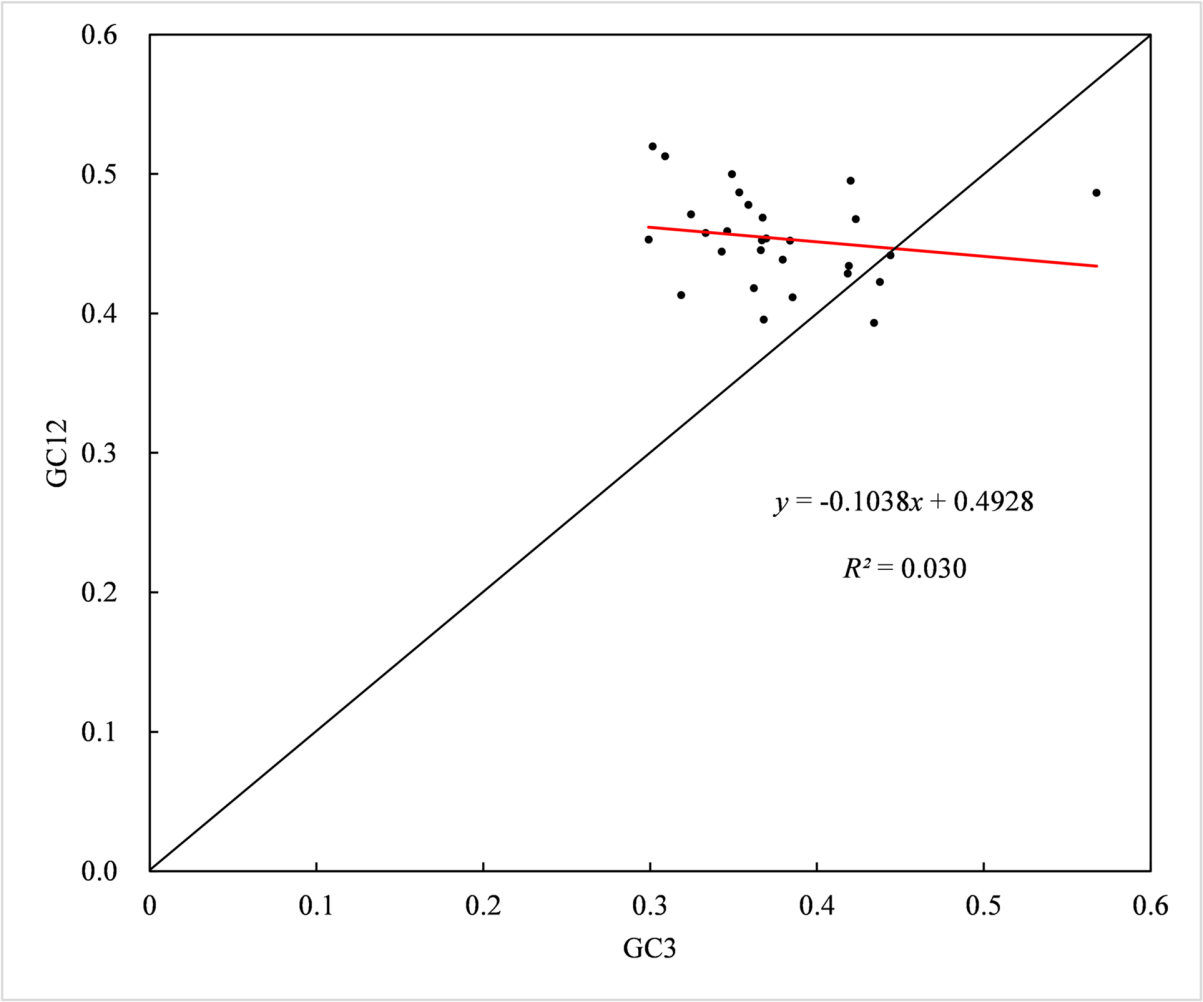
Neutrality plot analysis of the *H. citrina* mitogenome

To determine the relationship among bases at three codon positions, neutrality plot analysis was performed for each mt gene of *H. citrina* (Fig. 4). Narrow ranges of GC3 and GC12 (0.2991–0.5676 and 0.3933–0.5199, respectively) were observed, and only a few genes were diagonally distributed in the plot. Moreover, GC12 displayed no significant correlation with GC3 (*r*=-0.1755, *P* > 0.05), indicating that natural selection might have a considerable influence on the CUB of the *H. citrina* mitogenome. In addition, the slope of the regression line was − 0.1038, suggesting the mutation pressure effect accounted for only 10.38%. Consequently, the above results infer that natural selection is superior to mutation pressure in affecting the development of CUB in the *H. citrina* mitogenome.

**Fig. 5:**
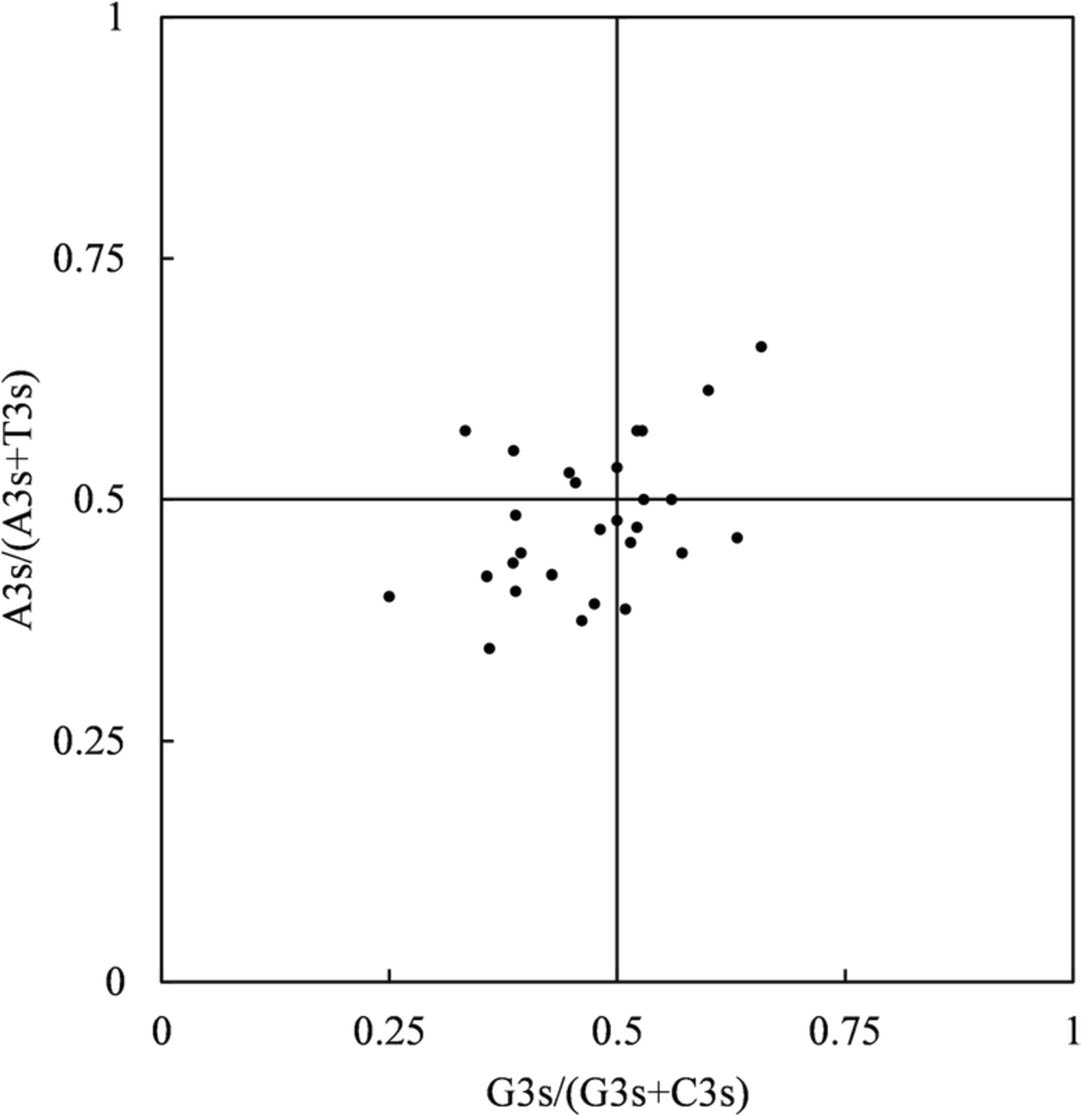
PR2-plot analysis of the *H. citrina* mitogenome

To further estimate the bias relationship of the four bases of mt genes, Parity rule 2 (PR2)**-**plot analysis was performed on the fourfold degenerate codon families. As depicted in Fig. 5, the distribution of genes is not uniform in the PR2-plane. Most of the points are in the lower half of the area along the vertical direction, revealing that the use frequency of T is higher than that of A at the synonymous position. However, in the horizontal direction, more genes are obviously distributed on the left side of the plane, so the content of C is higher than that of G. Consequently, higher levels of pyrimidines (T and C) are confirmed at the ‘silent’ site of the codon in the *H. citrina* mitogenome. The unbalanced usage of bases again illustrates that not only mutation but also selection and other factors determine the CUB patterns of the *H. citrina* mitogenome.

### Determination of RSCU values and putative optimal codons

**Fig. 6:**
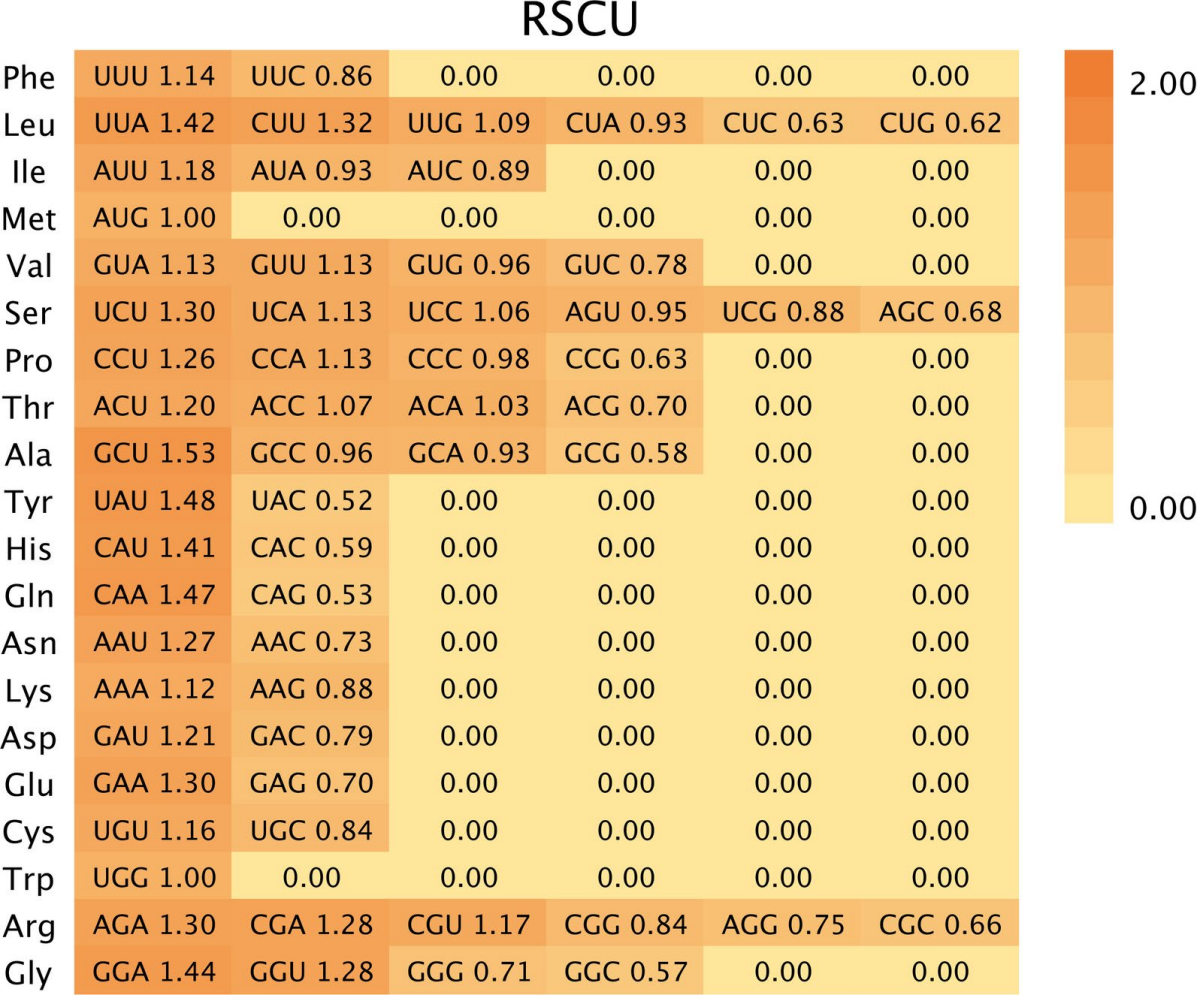
Heat map of codon usage preference based on RSCU values in the *H. citrina* mitogenome

In the present study, there were 29 codons with RSCU values greater than 1 defined as high-frequency codons (Fig. 6), indicating a high bias in the usage of these codons in the mitogenome of *H. citrina.* Excluding UUG (leucine), UCC (serine), and ACC (threonine), the remaining preferentially used codons end in A (11 of 29) or T (15 of 29). These results are further evidence that the mt gene of *H. citrina* is biased toward codons ending in A/T, illustrating that compositional constraints might have an impact on the synonymous CUB patterns of the *H. citrina* mitogenome.

By comparing the RSCU values from the two bias gene groups constructed by the ENC difference, 22 optimal codons were identified whose RSCU values were greater than 1 with ΔRSCU > 0.08 (Table 3). In the preferred codons, 19 codons ended with A (7/19) or T (12/19), while only three codons ended with G (2/3) or C (1/3). These results illustrate that both the high-frequency and optimal codons of the mt genes in *H. citrina* tend to end in A/T.

**Table 3:** RSCU of genes and the optimal codons of the mitogenome in *H. citrina*

Amino acid	Codon	RSCU _High_	RSCU _Low_	ΔRSCU	Amino acid	Codon	RSCU _High_	RSCU _Low_	ΔRSCU
Ala (A)	GCA^a^	1.14	0.84	0.31	Pro (P)	CCA	0.84	1.33	-0.49
	GCC^a^	1.43	0.84	0.59		CCC	0.95	1.00	-0.05
	GCG	0	0.47	-0.47		CCG	0.95	0.83	0.11
	GCU	1.43	1.86	-0.43		CCU^a^	1.26	0.83	0.43
Cys (C)	UGC	0	0.71	-0.71	Gln (Q)	CAA^a^	1.63	1.50	0.13
	UGU^a^	2.00	1.29	0.71		CAG	0.38	0.50	-0.13
Asp (D)	GAC	0.44	0.90	-0.46	Arg (R)	AGA	0.60	1.35	-0.75
	GAU^a^	1.56	1.10	0.46		AGG	0.60	1.01	-0.41
Glu (E)	GAA^a^	1.68	1.44	0.24		CGA^a^	1.60	1.08	0.52
	GAG	0.32	0.56	-0.24		CGC	0.40	0.94	-0.54
Phe (F)	UUC	0.48	0.97	-0.48		CGG	1.00	0.74	0.26
	UUU^a^	1.52	1.03	0.48		CGU^a^	1.80	0.88	0.92
Gly (G)	GGA^a^	1.48	1.08	0.40	Ser (S)	AGC	0.35	0.59	-0.24
	GGC	0.15	0.61	-0.46		AGU	1.06	1.12	-0.06
	GGG	0.30	1.22	-0.92		UCA	0.94	0.99	-0.05
	GGU^a^	2.07	1.08	0.99		UCC	1.18	1.12	0.06
His (H)	CAC	0.36	0.63	-0.26		UCG^a^	1.29	0.66	0.63
	CAU^a^	1.64	1.38	0.26		UCU	1.18	1.52	-0.34
Ile (I)	AUA	0.30	1.12	-0.82	Thr (T)	ACA	1.00	1.10	-0.10
	AUC	0.98	0.85	0.13		ACC	1.00	1.10	-0.10
	AUU^a^	1.73	1.04	0.69		ACG	0.60	0.94	-0.34
Lys (K)	AAA^a^	1.13	0.95	0.17		ACU^a^	1.40	0.86	0.54
	AAG	0.88	1.05	-0.17	Val (V)	GUA	0.95	1.17	-0.22
Leu (L)	CUA	0.68	0.83	-0.15		GUC	0.76	1.11	-0.35
	CUC	0.41	0.71	-0.30		GUG	0.19	0.92	-0.73
	CUG	0.14	0.53	-0.40		GUU^a^	2.10	0.80	1.30
	CUU^a^	1.23	1.13	0.10	Trp (W)	UGG	1.00	1.00	0
	UUA^a^	2.05	1.60	0.44	Tyr (Y)	UAC	0.13	0.64	-0.51
	UUG^a^	1.50	1.19	0.31		UAU^a^	1.87	1.36	0.51
Asn (N)	AAC	0.89	0.54	0.35	Met (M)	AUG	1.00	1.00	0
	AAU	1.11	1.46	-0.35					

Optimal codons are presented with^a^

#### Cluster and phylogenetic analyses

**Fig. 7:**
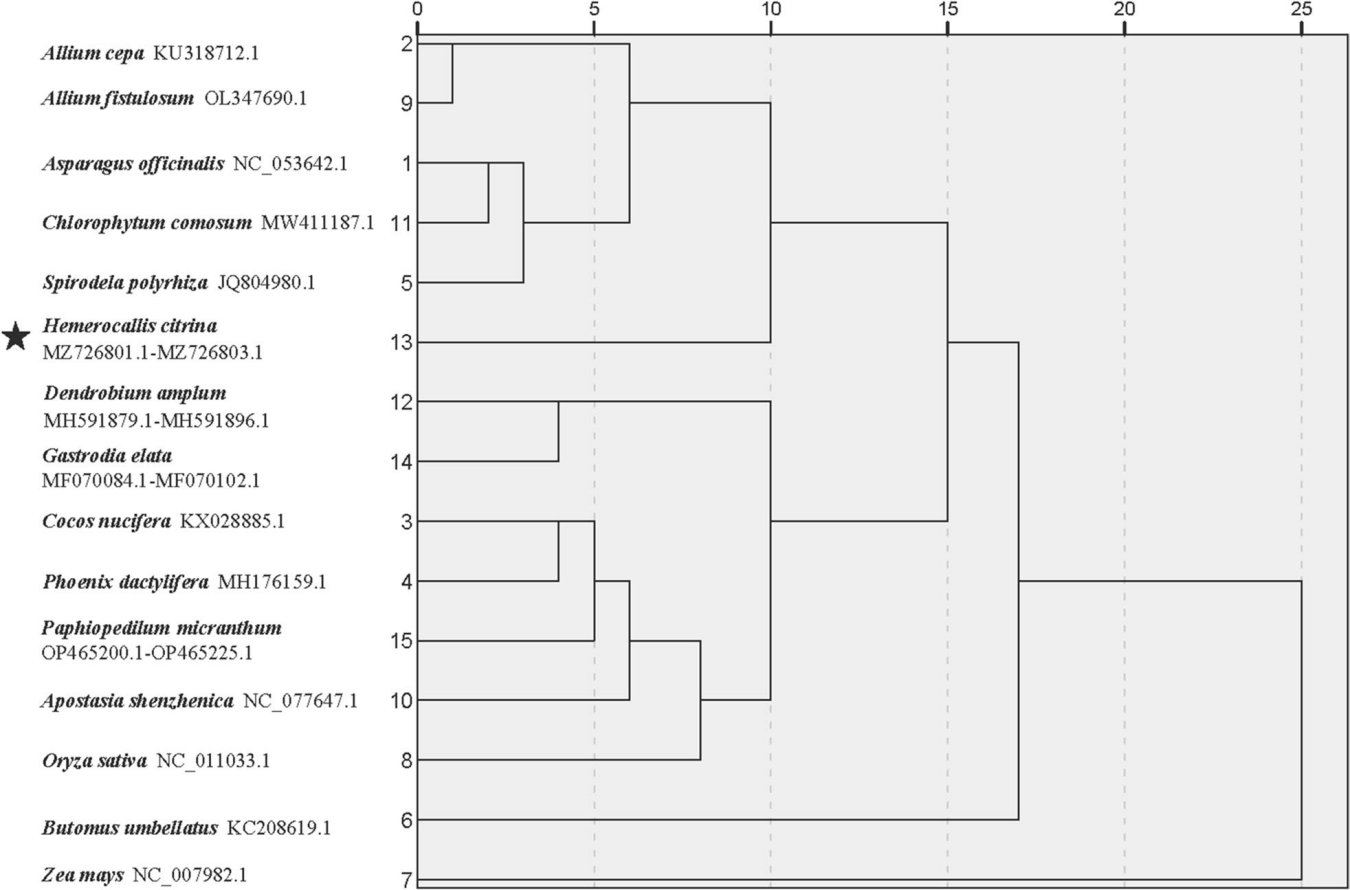
Clustering lineage plot based on RSCU values of the 15 monocotyledonous mitogenomes

**Fig. 8:**
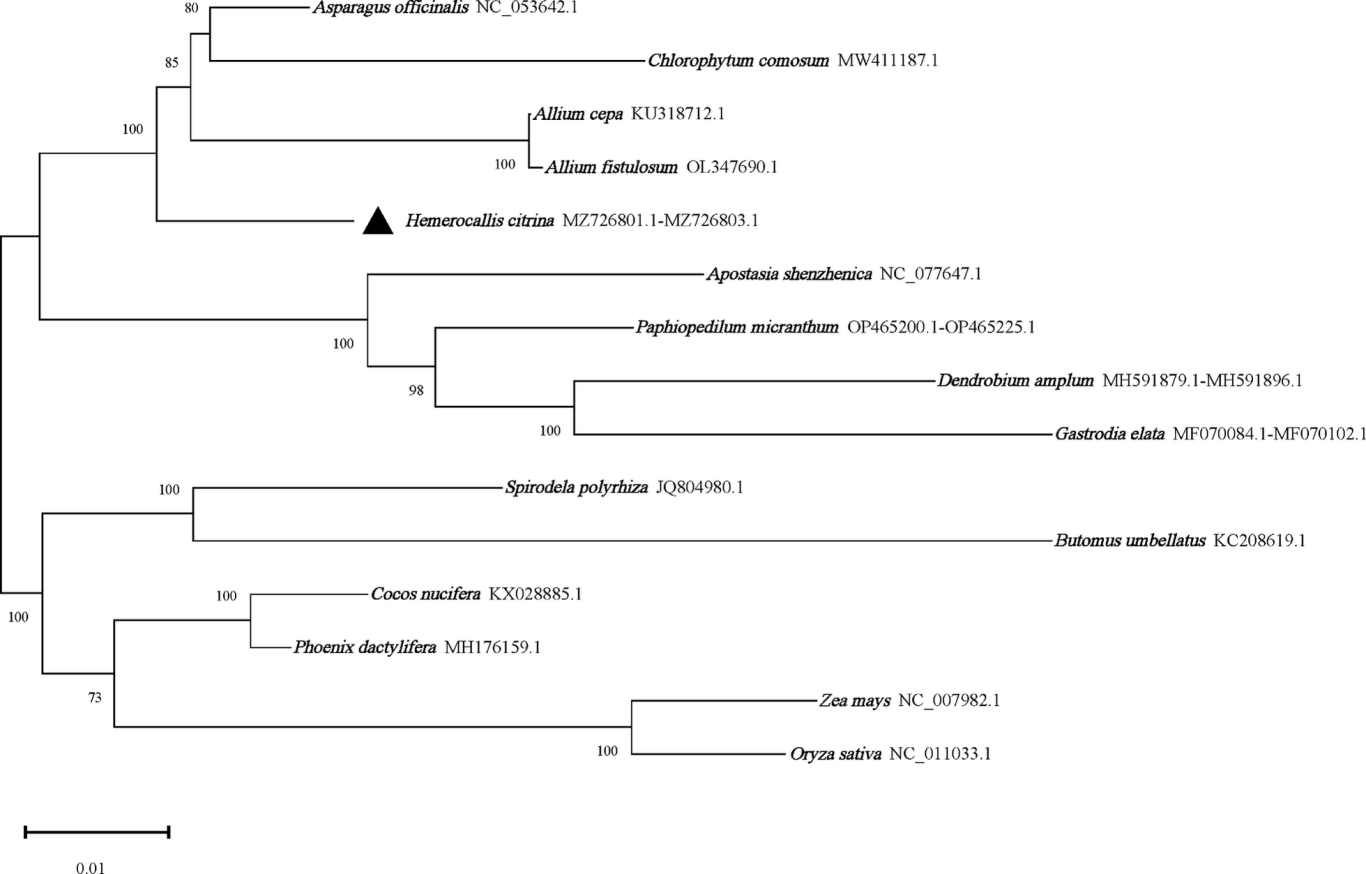
Phylogenetic tree based on the mt PCG for 15 monocot species

In order to gain a more accurate understanding of the divergence in the mitogenome codon usage, RSCU-based cluster analysis was conducted between *H. citrina* and other relatives. Since *H. citrina* is the only member of the Asphodelaceae family to have its complete mitogenome sequenced, 14 other monocotyledonous species with published mitogenome data were selected for subsequent comparison, i.e., *Asparagus officinalis* L. and *Chlorophytum comosum* (Thunb.) Baker of Asparagaceae, *Allium cepa* L. and *Allium fistulosum* L. of Amaryllidaceae, *Apostasia shenzhenica* Z.J.Liu & L.J.Chen, *Paphiopedilum micranthum* T. Tang & F. T. Wang, *Gastrodia elata* Blume, and *Dendrobium amplum* Lindl. of Orchidaceae, *Cocos nucifera* L. and *Phoenix dactylifera* L. of Arecaceae, *Zea mays* L. and *Oryza sativa* L. of Poaceae, *Spirodela polyrrhiza* (L.) Schleid. of Araceae, and *Butomus umbellatus* L. of Butomaceae. The RSCU-based cluster analysis results indicated that the analyzed monocotyledons group into two clusters (Fig. 7). The first cluster is a separate branch of *Z. mays*, while the second cluster is composed of the remaining 14 monocots. *H. citrina* along with *Allium cepa*, *Allium fistulosum*, *Asparagus officinalis*, *Chlorophytum comosum*, and *S. polyrrhiza* are classified as one clade, indicating that these species have similar codon usage patterns. In addition, the phylogenetic tree based on the mt PCG was also established for validation. As seen in Fig. 8, although the 15 analyzed species are samely divided into two clades, there are several differences between the topologies of the two graphs, at least when distant taxa are compared. The analyzed Arecaceae and Orchidaceae plants were classified into different clades of the phylogeny. While *Cocos nucifera* and *Phoenix dactylifera*, which belong to Arecaceae share a similar RSCU with Orchidaceae taxa (*Paphiopedilum micranthum* and *Apostasia shenzhenica)*. *Z. mays* and *O. sativa*, both members of the Poaceae family, were more distantly related in the RSCU-based clustering lineage. *H. citrina* clusters together with *Asparagus officinalis*, *Chlorophytum comosum*, *Allium cepa*, and *Allium fistulosum*, which intensely indicates their close relationships in evolutionary terms. When more closely related species are considered, such as *H. citrina* and Asparagaceae and Amaryllidaceae, a similar codon usage preference is observed. Consequently, *H. citrina* is close to *Asparagus officinalis*, *Chlorophytum comosum*, *Allium cepa*, and *Allium fistulosum* in evolutionary terms, reflecting a certain correlation between CUB and evolutionary relationships. These findings further support the likelihood that species with a close evolutionary relationship might have more similar codon usage preferences. However, it is worth noting that the position of *S. polyrrhiza* in the cluster analysis is quite different from that of the phylogenetic tree. The mt PCG-based phylogenetic tree is closer to the true evolutionary classification of the 15 monocotyledonous species. The discrepancy of taxonomic characters illustrates that the loci mutation of the genome sequence also plays an important role in the evolution of organisms.

## Discussion

Codon usage bias (CUB) in genomes is inevitable and refers to the uneven use of synonymous codons in gene coding to account for both gene regulation and molecular evolution. Previous studies have focused on the CUB patterns in many prokaryotes and eukaryotes, which was found to differ across various species and genes [10, 11]. The ancestors of terrestrial plants are believed to be unicellular algae, which have undergone a prolonged period of selection favoring the enrichment of GC in their nuclear genomes [35]. However, the CUB of the cp and mt genomes differ from their host cell counterparts in terms of evolutionary rates and patterns [36]. It has been proposed that organellar genes exhibit AT-richness and bias toward A- or T-ending codons in their genomes [37–39]. Extensive studies on the codon preference of the cp genomes have been published for a wide variety of organisms, for instance, *Oryza* plants [40], *Elaeagnus* plants [41], *Epimedium* plants [42], Euphorbiaceae species [39], Asteraceae species [43], and Theaceae species [44], among others. Nevertheless, the status of plant mitogenomes has not been well surveyed. Here, we conducted comprehensive analysis on the CUB of the mt genes in *H. citrina*. Composition analysis of codons revealed that the GCall and GC3 of the mt genes were lower than 50%, presenting a preference for A/T-rich nucleotides and A/T-ending codons in *H. citrina*. Moreover, the high-frequency and optimal codons in the *H. citrina* mitogenome are predominantly A/T-ending codons. Similar findings have also been recorded in previous studies on the mitogenomes of *O. sativa* [45], *Triticum aestivum* L., *Z. mays*, *Arabidopsis thaliana* (L.) Heynh., and *Nicotiana tabacum* L. [37]. Our results lend further support to the evidence that the GC composition is the factor that most directly reflects the CUB patterns.

Investigations of the factors influencing CUB in genomes have been continuous since striding into the era of genomics research. Various hypotheses have been proposed toward unraveling the reasons for deviations in CUB. Two typically accepted hypotheses explaining the origin of CUB are the selection–mutation–drift model [46] and neutral theory [47]. Ultimately, although CUB is determined by various factors, it appears that the evolution of CUB is a primary result of the balance between natural selection and directional mutation pressure. Research on *Helianthus annuus* L. suggests that mutation pressure is the most dominant evolutionary driving force of the cp genome [48]. However, in most cp genomes, natural selection would be more prominent in the formation of codon usage patterns [39–42]. With regard to plant mitogenomes, natural selection is considered to be the crucial factor shaping CUB [37, 45]. In our present study, only a few genes approached the expected curve, whereas most genes were discretely distributed in the ENC-plot, implying mutation pressure is a minor factor of CUB. Combined neutrality plot and PR2-plot analyses augment the inference that the CUB of the *H. citrina* mitogenome are attributed to natural selection and mutation pressure, while natural selection is the decisive factor. Moreover, we found significant correlations of ENC with the GC3 and codon counts, suggesting that not only compositional constraints but also gene length contributes greatly to CUB. Therefore, we conclude that not only mutation but also selection and other factors, in combination, significantly contribute to framing the CUB patterns of the *H. citrina* mitogenome, and natural selection is the main determinant.

The diversity of the CUB among various organisms can provide valuable information for species classification and molecular evolution. Research has indicated that there is a certain correlation between the distance of genetic relationships within species and codon usage preferences [22]. Here, we performed RSCU-based cluster analysis between *H. citrina* and 14 other monocots. *H. citrina* along with *Allium cepa*, *Allium fistulosum*, *Asparagus officinalis*, *Chlorophytum comosum*, and *S. polyrrhiza* were classified as one cluster, indicating that they share similar codon usage patterns. The phylogenetic tree, subsequently established based on the mt PCG, confirmed that *H. citrina* is evolutionarily close to *Asparagus officinalis*, *Chlorophytum comosum*, *Allium cepa*, and *Allium fistulosum.* Our findings are quite consistent with research on the cp genome of *Mesona chinensis* Benth [22], displaying a certain correlation between CUB and the evolutionary relationships. However, the phylogenetic relationship of the nuclear genomes between cotton species cannot be well reflected by taxonomic results based on codon RSCU values [49]. The likely explanation is the fairly weak codon usage preference in the *H. citrina* mitogenome, and thus the mt genes are not susceptible to external factors during evolution. Consequently, RSCU-based cluster analysis can complement taxonomic studies of *H. citrina.* Nevertheless, it is worth noting that the position of *S. polyrrhiza* in the cluster analysis is quite different from that of the phylogenetic tree. These results further indicate that the evolutionary relationship based on codon preference characteristics may miss some useful information, such as the non-preference codon information in CDS, which indirectly demonstrates that the non-preference codons also play an important role in organism evolution and phylogeny.

For mitogenomes, although there are tremendous variations in the size, structure, and sequence among different species, the products encoded by mt genes are quite conservative [24]. Codon usage preferences affect gene expression through the preferential use of optimal codons to regulate the translational accuracy and efficiency [37]. Therefore, an investigation of CUB in the mitogenome could provide a basic understanding of mitogenomic evolution and offer deeper insight into improving the expression efficiency of exogenous target genes in host organisms. Typically, the optimal genes in the nuclear genome use predominantly C- or G-ending codons, whereas those in the organelle genome prefer A- or T-ending codons [37, 50, 51]. In this study, we identified a total of 29 high-frequency codons and 22 optimal codons, and most of them exhibit a preference for A or T at the synonymous site. Notably, the mitogenomes of higher plants such as *T. aestivum*, *N. tabacum*, *Arabidopsis thaliana*, *Z. mays*, *Phycomitrella patens*, and *Marchntia polymorpha* also tend to have optimal codons that end in A or T [37]. The optimization of codons will contribute essential information for the genetic transformation and protein expression of mt genes in *H. citrina*.

## Materials and methods

### Sequence retrieval

The mitogenome sequences of *H. citrina* (MZ726801.1、MZ726802.1, and MZ726803.1) were retrieved from the National Center for Biotechnology Information (NCBI) database (https://www.ncbi.nlm.nih.gov/nuccore/?term=Hemerocallis%20citrina%20mitochondrion). We extracted the CDS of the mitogenome that started with ATG and ended with TAG, TGA, or TAA. Each CDS was greater than 300 bp in length and had exact multiples of three in the base number. In addition, the sequences used for the subsequent analysis were processed by eliminating duplicate sequences and sequences containing ambiguous bases, i.e., other than A, C, G, and T.

### Analysis of codon usage characteristic parameters

The codon usage indicators of the selected CDS were analyzed using the CodonW v1.4.2 program (http://codonw.sourceforge.net/), including CAI, CBI, Fop, RSCU, GC3s, A3s, T3s, C3s, and G3s. The other codon composition indices, including ENC, GCall, GC1, GC2, and GC3, were determined using the online Cusp and Chips programs from EMBOSS (http://www.bioinformatics.nl/emboss-explorer/). Then, correlation analysis of the main characteristic parameters was performed using the Correlation Plot tool in Origin 2022 software based on the Pearson correlation coefficient method.

### ENC-plot analysis

ENC is a vital indicator to evaluate the degree of preference for the imbalanced use of synonymous codons [52]. Usually, the ENC value ranges from 20 to 61 and is negatively correlated with codon preference. A smaller ENC value indicates a gene with a stronger bias, thus displaying extreme preference of using a unique codon to individually encode each amino acid. Conversely, a gene with an ENC value higher than 35 is considered to have weak usage preference and even no bias in the case of an ENC value up to 61 [49]. GC3s represents the average GC content at the ‘silent’ site of synonymous codons and is an important index to reveal the nucleotide composition bias. The ENC-plot was compiled using the ENC value of each gene as the ordinate and GC3s as the abscissa to explore the decisive factor influencing CUB. The standard curve was drawn according to the following equation: $\text{ENC}_{\text{expected}}=2+\text{GC3s}+\frac{29}{\text{GC3s}+\left(1-\text{GC3s}\right)^2}$ [52]. Under the condition that mutation pressure is the sole determinant of codon usage, the genes are located on or close to the standard curve, whereas when the points fall below and are far away from the excepted curve, this suggests that natural selection and other factors may greatly affect codon bias [53]. In order to better evaluate the difference between the expected and actual ENC values, the ENC ratio was calculated following the previously described formula: $\text{ENC}_{\text{ratio}}=\frac{\text{ENC}_{\text{expected}}-\text{ENC}_{\text{actual}}}{\text{ENC}_{\text{expected}}}$ [50].

### Neutrality plot analysis

Neutrality plot analysis is commonly applied to study the correlation among bases at three codon positions, revealing the role of natural selection and mutation pressure in the CUB patterns [54, 55]. In the current neutral graph, an individual mt gene of *H. citrina* is represented by a discrete point. The mean value of GC1 and GC2 for each gene was denoted by GC12, and GC12 and GC3 serve as the respective ordinate and abscissa of the scatterplot. It was assumed that if a notable correlation exists between GC12 and GC3, namely, that the discrete points are diagonally distributed in the plot with a slope close to one, this indicates that the CUB is dominated by mutation pressure. Contrastingly, a regression curve with a slope of zero and no significant correlation between GC12 and GC3 imply pure natural selection [56].

### PR2-plot analysis

In previous studies, the development of codon usage patterns was confirmed to be associated with the base composition at the ‘silent’ site of the codon [57]. PR2-plot analysis is extensively applied to evaluate the bias relationship between A/T and C/G at the synonymous site of the codon and, further, to determine the effects of mutation, selection, or other factors on CUB. The analysis is particularly meaningful for amino acids of a coding gene with four synonymous codons [58]. Consequently, the plan scatter diagram was constructed with A3s/(A3s + T3s) as the ordinate and G3s/(G3s + C3s) as the abscissa. The four-codon amino acids, i.e., valine, proline, threonine, alanine, and glycine, were selected to calculate the composition frequency of the third base position of each gene. The center point of the plot represents A = T and G = C with both coordinates equal to 0.5, presenting that codon bias is entirely caused by mutation; otherwise, natural selection and other factors may act on codon preference. The degree of distribution deviation from the center allows us to determine the direction and degree of the base deviation [58].

### Analysis of RSCU and putative optimal codons

The RSCU value of a codon refers to the ratio between the observed usage value and the expectation, reflecting the relative usage preference for specific codon compositions encoding the same amino acid [59]. When RSCU is equivalent to 1, codon usage is unbiased, and the codon is therefore selected randomly or equally. Codons with RSCU values greater than 1 are taken as high-frequency codons, which illustrates that codon usage is biased with high preference; the converse indicates the specific codon frequency is low [60]. For high-frequency codons, the codon whose ENC difference exceeds a certain critical value is determined to be an optimal codon [61]. The optimal codon is the preferred codon identified by calculating and ordering the ENC values of all genes. In general, highly expressed genes represent a large degree of codon preference and thus a small ENC value. On the basis of the above principles, 10% of the genes at the high and low end of the ordered ENC values were selected to establish low- and high-bias gene groups, respectively. The difference between the RSCU values of the codons from the two groups was calculated as ΔRSCU. The codons with RSCU > 1 and ΔRSCU > 0.08 were defined as the optimal codons of the gene [62].

### Clustering of codon usage preference and phylogenetic analyses

To explore the degree of divergence in the mitogenome codon usage more accurately, a cluster analysis was conducted between *H. citrina* and 14 other monocotyledons using SPSS 25.0 software. In the clustering process, each monocotyledon was taken as an object, and the RSCU values corresponding to 59 codons (excluding the codon AUG encoding methionine, UGG encoding tryptophan, and the three stop codons UAA, UAG, and UGA) were used as variables. The cluster pedigree was then established based on the squared Euclidean distance method [63]. Meanwhile, a contiguous sequence was constructed by lining up the 16 conserved mt PCGs (*atp1*, *atp6*, *atp9*, *ccmB*, *ccmC*, *ccmFc*, *ccmFn*, *cob*, *cox3*, *matR*, *nad3*, *nad4L*, *nad6*, *nad7*, *nad9*, and *rps12*) followed by alignment using MAFFT v.7.4.0 program [64] for the analyzed species. The maximum likelihood (ML) phylogenetic tree was constructed based on a Tamura-Nei model using MEGA 7 software [65] with 1,000 bootstrap replicates.

## Conclusions

In this study, mt genes of *H. citrina* were systematically analyzed to study the CUB patterns as well as the related forces influencing their evolutionary processes. The mitogenome exhibited weaker CUB and a preference for A/T-rich nucleotides and A/T-ending codons. Extensive measures were applied to evaluate the causes of CUB, as illustrated by the estimate of the codon usage characteristic indices, correlation, ENC-plot, neutrality plot, and PR2-plot analyses. Based on these, the formation of the CUB patterns of the *H. citrina* mitogenome is attributed to the combined effects of multiple factors, with natural selection being the decisive factor. Meanwhile, the RSCU-based cluster analysis and mt PCG-based phylogenetic tree revealed a certain correlation between CUB and evolutionary relationships. The inferred optimal codons also provide essential information for optimizing gene expression in *H. citrina*. In summary, these findings enrich our knowledge on the codon usage patterns of mitogenomes and serve as a fundamental reference for further studies on genetic modification and phylogenetic evolution in *H. citrina*.

## Data Availability

The mitochondrial genome datasets generated and analyzed in this study are available in the NCBI, *Hemerocallis citrine* (MZ726801.1-MZ726803.1, https://www.ncbi.nlm.nih.gov/nuccore/?term=Hemerocallis%20citrina%20mitochondrion), *Allium cepa* (KU318712.1, https://www.ncbi.nlm.nih.gov/nuccore/KU318712.1), *Allium fistulosum* (OL347690.1, https://www.ncbi.nlm.nih.gov/nuccore/OL347690.1), *Apostasia shenzhenic*a (NC_077647.1, https://www.ncbi.nlm.nih.gov/nuccore/NC_077647.1), *Asparagus officinalis* (NC_053642.1, https://www.ncbi.nlm.nih.gov/nuccore/NC_053642.1), *Butomus umbellatus* (KC208619.1, https://www.ncbi.nlm.nih.gov/nuccore/KC208619.1), *Chlorophytum comosum* (MW411187.1, https://www.ncbi.nlm.nih.gov/nuccore/MW411187.1), *Cocos nucifera* (KX028885.1, https://www.ncbi.nlm.nih.gov/nuccore/KX028885.1), *Dendrobium amplum* (MH591879.1-MH591896.1, https://www.ncbi.nlm.nih.gov/nuccore/?term=Dendrobium+amplum+mitochondrion%2C+complete+genome), *Gastrodia elata* (MF070084.1-MF070102.1, https://www.ncbi.nlm.nih.gov/nuccore/?term=Gastrodia%20elata%20chromosome%20mitochondrion%2C%20complete%20sequence), *Paphiopedilum micranthum* (OP465200.1-OP465225.1, https://www.ncbi.nlm.nih.gov/nuccore/?term=Paphiopedilum%20micranthum%20chromosome%20mitochondrion%2C%20complete%20sequence), *Phoenix dactylifera* (MH176159.1, https://www.ncbi.nlm.nih.gov/nuccore/MH176159.1), *Spirodela polyrrhiza* (JQ804980.1, https://www.ncbi.nlm.nih.gov/nuccore/JQ804980.1), *Oryza sativa* (NC_011033.1, https://www.ncbi.nlm.nih.gov/nuccore/NC_011033.1), and *Zea mays* (NC_007982.1, https://www.ncbi.nlm.nih.gov/nuccore/NC_007982.1).

## Abbreviations

A: Adenine
C: Cytosine
CAI: Codon adaptation index
CBI: Codon bias index
CDS: Coding sequences
Cp: Chloroplast
CUB: Codon usage bias
ENC: Effective number of codons
Fop: Frequency of optimal codons
G: Guanine
GCall: The overall GC content of the genome
GC1, GC2, and GC3: The GC content at each codon position
GC12: The average value of GC1 and GC2 for each gene
GC3s: The average GC content at the third position of synonymous codons
Mt: Mitochondrial
NCBI: National Center for Biotechnology Information
PCGs: Protein coding genes
PR2: Parity rule 2
RSCU: Relative synonymous codon usage
T: Thymine
T3s, A3s, C3s, and G3s: The frequency of T, A, C, and G at the third position of synonymous codons
U: Uracil
